# Effect of Flecainide in Idiopathic Premature Ventricular Contractions and the Induced Cardiomyopathy—UNIFLECA: A Single Arm, Non-Randomized Trial: Review of the Literature and Initial Results

**DOI:** 10.3390/jpm15040132

**Published:** 2025-03-29

**Authors:** Sotirios Kotoulas, Dimitrios Tsiachris, Michail Botis, Athanasios Kordalis, Dimitrios Varvarousis, Georgios Leventopoulos, Eleftherios Kallergis, Ioannis Doundoulakis, Leonidas E. Poulimenos, Konstantinos Tsioufis

**Affiliations:** 1 First Department of Cardiology, National and Kapodistrian University, “Hippokration” Hospital, GR11527 Athens, Greece; soter96@icloud.com (S.K.); mgmpotis94@gmail.com (M.B.); akordalis@gmail.com (A.K.); doudougiannis@gmail.com (I.D.); ktsioufis@gmail.com (K.T.); 2 Cardiology Department, Asklepeion General Hospital, GR16673 Athens, Greece; 3 Cardiology Department, University Hospital of Patras, GR26504 Patras, Greece; levent2669@gmail.com; 4 Cardiology Department, Heraklion University Hospital, GR71500 Crete, Greece; eleftherioskallergis@gmail.com

**Keywords:** PVC, flecainide, PVC-induced cardiomyopathy, heart failure, antiarrhythmics, PVC burden, Arrhythmology, idiopathic PVC, ventricular arrhythmias

## Abstract

**Background/Objectives**: Persistent high Premature Ventricular Contraction (PVC) burden (>10%) may result in PVC-induced cardiomyopathy. Current guidelines, supported by limited evidence, recommend flecainide for PVCs originating from the ventricular outflow tract (Class IIa). UNIFLECA is a prospective cohort study, aiming to assess the efficacy and safety of flecainide in PVC burden reduction in adults, irrespective of PVC origin, focusing secondarily on symptom relief and improvement of left ventricular ejection fraction (LVEF) in patients suffering from PVC-induced cardiomyopathy. **Methods**: Participants were adults with frequent PVCs, defined as PVC burden > 5%, confirmed by two 24 h Holter recordings taken at least one month apart, who denied catheter ablation treatment. Patients who were deemed ineligible for catheter ablation were also included. A total of 50 patients were screened and 35 were administered Flecainide, with dosage adjustment based on follow-up Holter results and QRS increases. Changes in PVC burden, LVEF, symptomatic status, along with treatment adherence, were evaluated. **Results**: In adults with frequent PVCs, flecainide led to a significant reduction in PVC burden, with a mean decrease of 76.2% in the first month, and 63.1% of patients achieving a PVC burden reduction greater than 80%. **Conclusions**: UNIFLECA contributes to the understanding of how personalized, non-interventional therapeutic modalities can be employed to manage PVCs, especially for patients unwilling to have or ineligible for ablation procedures.

## 1. Introduction

Premature ventricular contractions (PVCs) are a common finding in clinical practice. PVC-induced cardiomyopathy is defined as the sometimes-reversible deterioration of ventricular function due to the arrhythmia [1]. A well-established risk factor is a 24 h PVC burden greater than 10%. The decision of therapeutic management of the arrhythmia is determined by the presence of symptoms or the development of PVC-induced cardiomyopathy [1]. Treatment options include catheter ablation and pharmacological therapy, which comprises Class IC antiarrhythmics, beta-blockers, and calcium channel blockers [2,3,4,5,6]. Flecainide is a Class IC antiarrhythmic drug that acts as a potent inhibitor of the sodium channels of cardiac muscle fibers [7]. The aim of this study is to evaluate the effectiveness of flecainide in reducing the PVC burden, along with the reversal of the PVC-induced cardiomyopathy, while monitoring its safety and efficacy, recurrence rates, and compliance with its use. Potential prognostic factors for recurrence or non-compliance are also to be recorded.

## 2. Review of the Literature

### 2.1. Premature Ventricular Contractions

Premature ventricular contractions (PVCs) in the absence of structural heart disease or inherited ion channel diseases are defined as idiopathic. They are one of the most common arrhythmias encountered in daily clinical practice, with their impact seemingly increasing [1]. The exact mechanisms underlying idiopathic PVCs remain unknown, but possible mechanisms include triggered activity or microreentry. PVCs can originate from various sites within the heart, including both the endocardium and, less commonly, the epicardium [8].

Each PVC origin site produces a distinct electrocardiographic pattern. Anatomically, the most common sites of origin for PVCs include the right and left ventricular outflow tracts, aortic root, atrioventricular (AV) rings, and Purkinje fibers. Regarding PVCs originating from the right ventricular outflow tract (RVOT), various etiologic mechanisms have been proposed [9]. These include the preservation of the fetal conduction system in the right ventricle and subclinical myocarditis, undetectable with the current MRI techniques. PVCs originating from the aortic root are associated with the extensive fibrous tissue between the base of the aortic valve leaflets and the left ventricular myocardium. PVCs originating from the outflow tracts (right or left ventricular outflow tract) generally have higher treatment success rates with catheter ablation due to easier accessibility and distinct electrocardiographic features [10]. In contrast, PVCs arising from other areas, such as the papillary muscles or epicardial regions, tend to be more challenging to be treated successfully, due to their complex anatomical locations, requiring more advanced mapping techniques or multiple ablation attempts. For fascicular PVCs or ventricular tachycardia (VT), calcium channel blockers (CCBs) are recommended as the preferred treatment. Although evidence is limited, beta-blockers or CCBs are generally considered the first-line options for PVCs originating from areas other than the right ventricular outflow tract (RVOT) or left fascicles [10].

### 2.2. Prevalence

The PVC prevalence is proportional to the ECG recording duration. A two-minute electrocardiographic recording in the population of the ARIC (Atherosclerosis Risk in Communities) study revealed premature ventricular contractions (PVCs) in over 6% of the participants [11]. Ahn et al. reported a prevalence of 1–4% based on a 12-lead electrocardiogram and 40–75% based on 24 to 48 h Holter rhythm monitoring in the general population [12]. The increased frequency of PVCs in 24 h recording is associated with male sex, increased age, smoking, and increased values of systolic blood pressure [13].

### 2.3. Holter-Monitoring and PVCs

A 24 h Holter-Monitoring is the preferred method for diagnosing PVCs. Data from studies based on 24 h rhythm recordings are conflicting and variable. The fluctuation in PVC burden over a 24 h period is significant, as evidenced by 14-day recordings [14]. In a 15-year follow up of 55 patients with PVCs, Gaita et al. reported spontaneous remission of arrhythmia in 55% of participants [15]. In a pediatric population without structural heart disease, PVCs spontaneously resolved in 28% of patients, with a mean time to remission of 115.2 ± 6.4 months [16]. More recently, Lee et al. documented spontaneous remission in 44% of the population over a median follow up of 15.4 months [17]. Interestingly, Gatzoulis et al. recorded similar rates of satisfactory response over a period of 56.8 ± 43 months among symptomatic patients with idiopathic PVCs originating from the right ventricular outflow tract (RVOT), regardless of whether they received antiarrhythmic therapy (82%) or not (86%) [18].

### 2.4. Prognosis

PVCs have been associated with increased mortality in patients with ischemic heart disease. However, no causal relation between PVCs and increased mortality in the context of ischemic heart disease has been established, as the elimination of PVCs in myocardial infarction (MI) survivors has sometimes led to worse outcomes [19]. Dukes et al. demonstrated that a higher frequency of premature ventricular contractions (PVCs) is significantly associated with increased mortality. Participants in the highest quartile of PVC frequency had a 31% higher risk of death compared to those in the lowest quartile, even after adjusting for multiple confounding factors such as age, sex, BMI, and comorbidities. The study also found that PVCs contributed to mortality risk, in part through the development of congestive heart failure (CHF), explaining about 26.8% of this relationship. Additionally, the specificity of PVCs for predicting a 15-year risk of CHF was over 90% when PVCs comprised at least 0.7% of total ventricular beats, emphasizing their potential as a strong predictor of adverse outcomes. Given these findings, PVCs may represent an important and modifiable risk factor for both CHF and mortality, warranting further investigation into potential therapeutic interventions. Pay et al. demonstrated that PVCs occurring during treadmill exercise testing and the recovery phase have been associated with increased long-term mortality in patients without significant coronary artery obstruction [20]. In the Cardiovascular Health Study (CHS), 24 h rhythm monitoring was conducted on 1429 participants. Those in the highest quartile of PVC frequency exhibited a 31% increased risk of mortality and a 48% higher risk of developing heart failure compared to participants in the lowest quartile [21].

### 2.5. Diagnosis

Cardiac MRI has shed light on the prognosis of PVCs, particularly by distinguishing idiopathic PVCs from those related to structural heart disease (SHD). To classify PVCs as idiopathic, it is essential to exclude underlying SHD, typically accomplished through Cardiac Magnetic Resonance Imaging (CMR). CMR has been utilized to detect SHD in patients presenting with ventricular arrhythmias (VA) but normal findings on echocardiography. In a cohort of 946 patients with significant ventricular arrhythmia (PVCs > 10.000/24 h), CMR identified SHD in 25.5% of cases, revealing conditions including myocarditis, arrhythmogenic right ventricular cardiomyopathy (ARVC), dilated cardiomyopathy (DCM), and other cardiac abnormalities. The study highlighted that chest pain and sustained ventricular tachycardia were the strongest independent predictors of SHD on CMR imaging, demonstrating the utility of CMR in uncovering concealed structural abnormalities that could act as substrates for arrhythmias [22].

Similarly, another study involving 518 patients with PVCs, where SHD was excluded through routine diagnostic testing, Muser et al, reported that the patients with abnormal findings on cardiac MRI were at a higher risk for the composite endpoint of sudden cardiac death, preventable sudden cardiac death, and ventricular tachycardia or fibrillation [23]. Key risk factors for these adverse outcomes included male gender, a family history of sudden cardiac death or cardiomyopathy, and multifocal PVCs. Furthermore, patients with multifocal PVCs or morphologies other than left bundle branch block (LBBB) morphology had higher incidence of abnormal findings on MRI imaging. These findings emphasize that, although idiopathic PVCs are often considered benign, CMR can identify underlying structural heart disease, thereby enhancing risk stratification and guiding management for patients at greater risk of severe cardiac events.

### 2.6. Symptomatology

The asymptomatic nature of premature ventricular contractions (PVCs) is a primary factor contributing to their underdiagnosis [24]. The presence of symptoms such as palpitations, dizziness, and chest pain significantly impairs patients’ quality of life. PVCs often do not produce a palpable peripheral pulse, leading to potential clinical oversight. Trigeminy and bigeminy, which frequently occur at rest, can result in pseudo-bradycardia (true bradysphygmia) and contribute to a subjective sense of fatigue.

### 2.7. PVC-Induced Cardiomyopathy

Frequent premature ventricular contractions (PVCs) can impair left ventricular function, a condition that is often reversible following the elimination of PVCs [2,25,26]. The burden of PVCs is recognized as the most significant risk factor for the development of PVC-induced cardiomyopathy (PVC-CM). The threshold of PVC burden which is associated with the development of PVC-CM ranges from 16% to 26% [25,27]. PVC-CM is found in about 7% of patients with a PVC burden over 10%, though this is likely an underestimation [28]. Among those referred for PVC ablation, the prevalence of PVC-CM ranges between 9% and 30% [4,29]. According to the current ESC guidelines, a PVC burden of 10% or greater is considered the minimum threshold at which there is an increased risk for developing PVC-induced cardiomyopathy and thus treatment is required [10].

A systematic review and metanalysis of the risk factors for the development of PVC-CM revealed that additional factors associated with the development of PVC-CM include a QRS duration greater than 150 milliseconds, presence of non-sustained VT, the absence of symptoms, interpolated PVCs, male gender, and an epicardial origin of the arrhythmia. The elimination of PVCs often leads to improvement in myocardial function, even in the presence of underlying structural heart disease. However, an increased QRS duration during sinus rhythm is a poor prognostic factor for the recovery of myocardial function following the elimination of PVCs [23,30].

### 2.8. Treatment Modalities

Therapeutic options for idiopathic PVCs include catheter ablation and antiarrhythmic drug therapy (AAD).

Catheter ablation is highly effective for treating premature ventricular contractions (PVCs), with reported success rates ranging from 75% to 90%. It is considered the first-line therapy for PVC-induced cardiomyopathy. Factors influencing the immediate success of ablation and the clinical outcomes include the origin of the PVCs (with the highest success seen in outflow tract PVCs), the number of different PVC morphologies, and the absence of late gadolinium enhancement (LGE) on cardiac magnetic resonance (CMR) imaging [10,31]. Pharmaceutic modalities include Class I antiarrhythmics, beta-blockers, and non-dihydropyridine calcium channel antagonists [10].

In the context of increasing PVC load during exercise or at higher heart rates, the use of β-blockers is indicated, since they reduce symptoms by attenuating post-extrasystolic potentiation, which is explained via the Frank–Starling mechanism. However, at lower heart rates, β-blockers may not suppress PVCs, but actually worsen symptoms by enhancing pseudo-bradycardia [32,33]. Calcium channel antagonists are particularly effective against PVCs originating from the right or left ventricular outflow tracts [34].

The administration of Class IC antiarrhythmics is contraindicated in patients with structural heart disease or coexisting coronary artery disease, as evidenced by the Cardiac Arrhythmia Suppression Trial (CAST) [35]. Except for the CAST study, no other research has shown an increased risk of mortality in patients with PVCs without cardiac comorbidity. In patients with PVC-induced cardiomyopathy, Class IC antiarrhythmics, particularly flecainide and propafenone, effectively suppress PVCs and restore ejection fraction, challenging earlier data that associated these drugs with adverse events in patients with non-ischemic heart disease [36]. Amiodarone is preferred for patients with structural heart disease due to its efficacy, although its long-term use is associated with multiple adverse effects [37,38].

The safety and efficacy of Class IC antiarrhythmic drugs (AADs)—mainly flecainide but also propafenone, was studied in 34 patients with non-ischemic cardiomyopathy (NICM) and implantable cardioverter-defibrillators (ICDs), most of whom had previously failed other AADs or catheter ablation for PVCs. Their use led to a significant reduction in PVC burden from 20% to 6%, improved left ventricular ejection fraction (LVEF) from 33% to 37%, and increased biventricular pacing from 85% to 93%, while reducing episodes of sustained ventricular tachycardia and heart failure admissions [39].

### 2.9. Flecainide

Flecainide functions as a potent inhibitor of the Nav1.5 sodium channels in cardiac muscle fibers. Nav1.5 is a voltage-gated sodium channel predominantly located in cardiac muscle, crucial for the rapid upstroke of the action potential (phase 0), thereby initiating the “rapid depolarization” of cardiac myocytes. Flecainide targets the Nav1.5 channel in its inactivated state, diminishing the inward flow of sodium into the cell and delaying the normal transition of Nav1.5 from the inactivated to the closed conformation [7]. Consequently, flecainide’s effects on the heart include the prolongation of conduction across intra-atrial, nodal, and intraventricular pathways, as well as the extension of the effective refractory period, particularly in the atrioventricular node and accessory pathways, and, to a lesser extent, in the atria and ventricles.

Flecainide is approved for the treatment of premature ventricular contractions (PVCs). Even though it is endorsed by the ESC guidelines, the evidence supporting the use of flecainide is limited. Its administration is mainly indicated for symptomatic PVCs originating from the right ventricular outflow tract or a limb of the conduction system. A Class IIa recommendation is established for patients with preserved ejection fraction, while patients with reduced ejection fraction hold a Class IIb recommendation [10]. Flecainide is indicated for the treatment of PVC-induced cardiomyopathy, regardless of the arrhythmia’s origin. A large meta-analysis studying the safety of flecainide found that proarrhythmic events were significantly less frequent with flecainide compared to control groups (placebo, amiodarone, propafenone, sotalol, digoxin). Additionally, it revealed that treatment with flecainide was associated with a similar incidence of cardiac adverse events, except for a lower incidence of hypotension [40]. Regarding non-cardiac adverse events, flecainide exhibited significantly fewer gastrointestinal symptoms. Dizziness is the most common non-cardiac adverse event associated with flecainide, followed by blurred vision and difficulty focusing. These symptoms are typically mild, well-tolerated, and self-limiting, with visual disturbances often occurring on lateral gaze and lasting only a few seconds. Severe adverse events, including proarrhythmic effects, are uncommon when prescribed appropriately.

### 2.10. Current Data

The rationale for using flecainide in treating PVCs originates from the study of Abitbol et al., 1983 [41]. It is focused on the efficacy of flecainide in treating non-life-threatening premature ventricular contractions (PVCs) among 35 patients. The study concluded that a single dose of intravenous flecainide effectively suppressed 100% of PVCs in all patients for a time frame of 60 to 1440 min, with minimal and insignificant side effects.

The use and study of flecainide decreased significantly after the Cardiac Arrhythmia Suppression Trial (CAST) [35]. The effect of encainide and flecainide was examined to evaluate whether suppressing ventricular ectopy post-myocardial infarction reduces the incidence of sudden cardiac death. Their use was discontinued due to excessive mortality, attributed to episodes of sustained ventricular tachycardia post-acute myocardial infarction, prompting a re-evaluation of their use in this context. The study’s findings significantly contributed to understanding antiarrhythmic drug therapy in post-myocardial infarction patients, leading to changes in clinical practice and guidelines, while reducing the use of flecainide. Despite the initial discouraging results, contemporary evidence suggests a shift towards the use of flecainide in patients with coronary artery disease (CAD). Thereafter, several studies have assessed its safety, with a retrospective review of 348 patients showing no increase in mortality or ventricular arrhythmias in patients with either nonobstructive or obstructive CAD, indicating its potential safety in patients with stable coronary artery disease [31]. Ashraf et al. demonstrated flecainide’s safety in patients with stable nonobstructive CAD [39]. Overall, flecainide can be safe in carefully selected CAD patients.

In recent years, there has been a trend towards using Class I antiarrhythmics for treating PVCs. However, the available studies include a small number of patients, with the total number not exceeding 200.

In 2013, Zhong Li et al. found that Class I and III antiarrhythmic drugs were more effective than beta-blockers or calcium channel blockers in patients with more than 1000 PVCs per 24 h [42]. The average effectiveness of reducing PVCs with antiarrhythmic medication was approximately 49%, while the reduction with Class I and III antiarrhythmics was 70–90%. Flecainide was considered an effective choice for PVC suppression in the studied population.

Hwang et al. conducted a multicenter study including 500 patients with a PVC burden > 5% to evaluate the relative efficacy of ablation versus antiarrhythmic drugs in treating PVCs, focusing on their effect on reducing PVC burden and improving left ventricular systolic function [43]. Both treatments significantly reduced PVC frequency. Among patients receiving antiarrhythmic therapy, those treated with flecainide showed excellent results, with a PVC burden reduction of >80%.

In a recent study, flecainide was administered to 13 patients, resulting in a significant reduction in PVC burden and substantial improvement in left ventricular ejection fraction (LVEF), indicating effective PVC suppression and recovery of left ventricular systolic function in patients with suspected PVC-induced cardiomyopathy [36].

Bertels et al. studied flecainide use in younger patients (9–19 years old) [3]. The overall effectiveness of antiarrhythmic therapy in children with frequent PVCs was limited, except for flecainide, which showed significant PVC burden reduction, suggesting its potential as a more effective therapeutic option compared to beta-blockers, sotalol, or verapamil.

Kojic et al. conducted a single-center retrospective analysis comparing the efficacy and safety of flecainide, propafenone, and sotalol in treating symptomatic idiopathic PVCs. The primary outcome was achieving complete or near-complete PVC reduction, defined as >99% reduction in PVC burden [40]. The secondary outcome sought significant PVC burden reduction, defined as ≥80%. Flecainide demonstrated the highest achievement rate for both primary and secondary outcomes compared to propafenone and sotalol. Among treated patients, flecainide led to complete/near-complete PVC burden reduction in 56% and significant PVC burden reduction in 64% of cases. These findings support considering flecainide as the preferred choice in managing idiopathic PVCs when catheter ablation is not feasible or other first-line treatments have failed or are not tolerated.

In Pennsylvania, 34 patients with non-ischemic cardiomyopathy and ICDs were treated with flecainide (23/34 patients) and propafenone to evaluate their safety and efficacy. There was a significant reduction in PVC burden (from 20% to 6%) and improvement in LVEF (from 33% to 37%). The percentage of biventricular pacing increased from 85% to 93%. These improvements were consistent in patients treated with flecainide, demonstrating its effectiveness in suppressing PVCs and improving cardiac function in patients with non-ischemic cardiomyopathy (NICM) and ICDs. In carefully selected patients with NICM and protected by ICDs, Class IC antiarrhythmic drugs may be a viable therapeutic option [5].

The ECTOPIC trial, a randomized open-label cross-over study, evaluated the efficacy of flecainide compared to metoprolol in reducing premature ventricular contractions (PVCs) in pediatric patients. The study involved 19 children with a PVC burden greater than 15%, assessed through 48 h Holter recordings. Results indicated that flecainide significantly outperformed metoprolol, achieving a mean reduction in PVC burden of 10.6 percentage points compared to 2.4 percentage points for metoprolol. Additionally, nearly half of the patients treated with flecainide experienced a reduction in PVC burden below 5%, highlighting its superior effectiveness. The findings suggest that flecainide may be a more effective first-line treatment for symptomatic patients with frequent PVCs, in comparison with beta-blockers [6].

The available literature lacks long-term follow up and the duration of arrhythmia suppression is not explicitly recorded. Moreover, the total amount of patients that have received flecainide for the treatment of PVCs is less than 200, from 1983 to 2024, as shown in Table 1. Therefore, a large-scale study, focusing on patients receiving flecainide for the treatment of PVCs, is required. The UNIFLECA Study aims to address this critical research void.

### 2.11. The UNIFLECA Study

**Figure 1:**
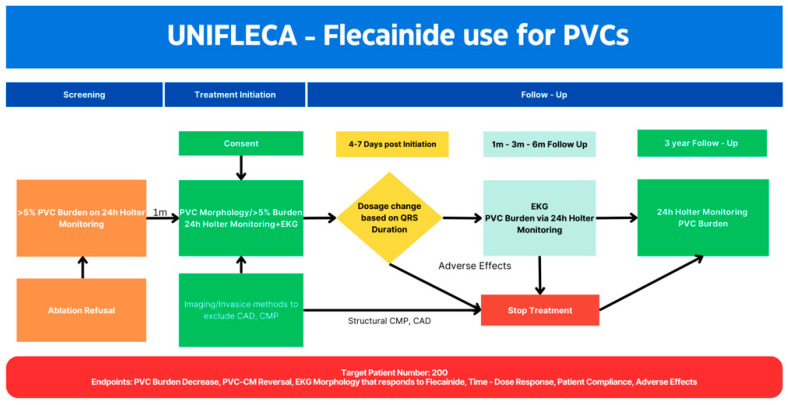
The UNIFLECA Study.

The efficacy of flecainide in reducing PVCs study is a prospective, multicenter study in tertiary Hospitals throughout Greece in an Arrhythmology outpatient setting. Patients presenting with increased PVC burden were recruited and afterwards screened for one month, prior to flecainide initiation. The follow up is scheduled to occur at three-month intervals over a total duration of three years (Figure 1).

## 3. Subjects and Methods

### 3.1. Inclusion/Exclusion Criteria

Patients need to fulfill the following inclusion criteria; 18 years of age or older and have an idiopathic PVC burden of over 5% in two 24 h Holter EKG recordings, 30 days apart, to account for any spontaneous remission of PVCs. The exclusion criteria include known intolerance or allergy to flecainide (n = 1); coronary artery disease with (n = 2) or without (n = 5) myocardial infarction (recent or old); heart failure; episode of sudden cardiac death; complete left bundle branch block; bifascicular block (n = 1); second or third-degree atrioventricular block; sinus node dysfunction and atrial cavernous disease without the presence of a pacemaker; long QT interval; hypertrophic cardiomyopathy; presence of other structural heart disease (n = 2); and pregnancy and lactation. As of September 2024, a cohort of 50 patients was screened. Among them, 35 patients met the eligibility criteria and exhibited a persistently high PVC burden, for which slow-release flecainide was initiated. (Median time between Holter recordings: 162 days, 6 patients had undergone an unsuccessful PVC ablation procedure; in 1 patient, PVCs re-occurred after an initially successful ablation attempt.) A total of 11 patients did not meet the inclusion criteria, while 2 patients opted for ablation therapy after the initial screening and 2 patients presented with spontaneous remission without any treatment.

### 3.2. Informed Consent and Ethical Approval

Patients were informed about the study in person and provided written informed consent. Ethical approval was granted from the local and central ethical committees.

### 3.3. Study Protocol

Baseline characteristics are collected, including sex, age, date of diagnosis and presence of symptoms, as well as other medications taken by the patient, with the use of a questionnaire in a case record form. Baseline testing consisted of a 12-lead ECG, echocardiography, 24 h Holter-recording, and an invasive or non-invasive method to exclude the presence of coronary artery disease. Cardiac MRI was supplied where available.

Eventually, an additional 24-h Holter-recording, within a time frame greater than 30 days, with persistent PVC burden of >5% was required. Flecainide was initiated at a dose of 100 mg. After 5 to 7 days, a 12-lead ECG was repeated and the flecainide dosage was adjusted, up to 200 mg per day, taking into consideration the alteration of the QRS duration.

A month after the initiation of the treatment, a repeated 24 h Holter-Monitoring was performed to assess the reduction in PVC burden, and the dosage of flecainide, respectively. The medication was stopped in patients with reduction of the PVC burden. During subsequent visits, patients and were asked about their experience of side effects.

Follow up was continued every 3 months with the use of 24 h Holter-Monitoring to assess the burden of PVCs along with a baseline ECG.

### 3.4. Outcome Parameters

The primary endpoint was defined as the reduction of PVC burden, expressed in percentage points, on a 24 h Holter-recording. This was determined by comparing the baseline PVC burden with the PVC burden recorded at the conclusion of the monitoring period. The co-primary endpoint was similarly quantified as the reduction in PVC burden on a 24 h Holter-recording, determined by comparing the baseline PVC burden with the PVC burden measured at the highest administered dosage.

## 4. Results

The study included patients aged >18 years old, with a male predominance (52.5%, n = 27). All participants were in sinus rhythm and had negative ischemia screening results. Left ventricular ejection fraction (LVEF) was reduced in 32.5% (n = 17) of the patients, ranging between 45 and 57%. The predominant symptoms reported were palpitations (70%, n = 35), dizziness (27.5%, n = 14), and tachycardia (17.5%, n = 9), while 20% (n = 10) of patients were asymptomatic. Notably, 12.5% (n = 6) had undergone prior failed ablation attempts, and 82% (n = 41) had received previous medication without achieving symptom control, primarily β-blockers including metoprolol, bisoprolol, and nebivolol. Cardiac risk factors were common in this cohort, including hypertension and dyslipidemia (47% each, n = 47), smoking (42%, n = 21), diabetes mellitus (20%, n = 10), and obesity (12%, n = 6). PVCs were observed, ranging from 5200 to 64.800 per 24 h, with a mean heart rate between 54 and 101 beats per minute. Non-sustained ventricular tachycardia (NSVT) was present in 77.5% (n = 39) of cases, and 60% (n = 30) of patients exhibited ventricular couplets. The majority (90%, n = 45) of PVCs were monomorphic, though some patients demonstrated polymorphic beats with two distinct morphologies.

A daily dose of 100 mg slow-release flecainide was administered in 63.2% (n = 22) of patients, while the remaining patients (n = 13) received a 200 mg daily regimen. Among them, 19 were monitored over a one-month period following treatment initiation. A significant reduction was present in the mean PVC burden (mean decrease: 76.2% in the first month), with 63.1% (n = 12) of patients achieving a PVC reduction greater than 80%. Symptomatic improvement was observed in 74% (n = 14) of patients, with 25.8% (n = 5) reporting complete resolution of symptoms. Among those with baseline left ventricular ejection fraction (LVEF) impairment, functional improvement was noted, and 68% (n = 13) required dose adjustments to achieve optimal efficacy. No patients exhibited a QRS increase of >25% and no major adverse effects were presented. Beyond the significant reduction in PVC burden, patients reported improvements in quality-of-life metrics, such as reduced fatigue and better exercise tolerance. The study has certain limitations as it is a single arm, non-randomized, controlled study and the number of patients included is smaller than the calculated sample size; however, the study remains ongoing. This investigator-initiated study has been delayed due to the extended screening period and the occurrence of spontaneous PVC remission.

## 5. Conclusions

Flecainide has demonstrated significant efficacy in decreasing the burden of premature ventricular contractions (PVCs) while improving symptoms and functional status in adults with persistent high PVC burden. It serves as a valuable therapeutic alternative for patients who are either unsuitable for or unwilling to undergo catheter ablation, regardless of PVC origin. Furthermore, real-world implementation of flecainide-based therapy should be considered in diverse patient populations, including those with limited access to advanced diagnostics or ablation facilities. What is more, the improvement of quality-of-life-metrics underscores the unmet need to include patient-reported outcomes into therapeutic evaluation when it comes to PVC pharmacological therapy.

These findings align closely with the evolving focus on precision cardiology. By leveraging advanced diagnostic tools such as cardiac MRI, clinicians can better visualize structural anomalies and identify arrhythmic substrates. This tailored approach enables the refinement of patient selection, ensuring optimal outcomes while minimizing risks.

The use of flecainide for PVCs exemplifies the potential of personalized medicine by incorporating patient-specific data, individual values, and personal preferences, alongside advanced diagnostics to enhance both therapeutic efficacy and safety.

## Figures and Tables

**Table 1:** Studies using flecainide in treatment of PVCs.

Study	Year	Number of Patients on Flecainide	Inclusion Criteria	Results—Burden Reduction
Bertels et al.—ECTOPIC (<18 y.o.) [3]	2024	18	>15% PVC burden	>95%
Mohammad Raad et al. [5]	2024	23	>10% PVC burden, non-ischemic cardiomyopathy with ICD	80%
Hwang et al. [43]	2023	52	>5% PVC burden	>60%
Kojic et al. [40]	2023	58	>5% PVC burden	>80% in 64% of patients >99% in 56% of patients
Bertels et al. [3]	2021	10	>5% PVC burden (<18 years old)	>45%
Hyman et al. [36]	2017	20	>7.5% PVC burden	90%
Zhong Li et al. [42]	2013	9	>1000 PVCs in 24 h	83%
Abitbol et al. [41]	1983	35	>600 PVCs in 24 h	>50%

## Data Availability

The original contributions presented in this study are included in the article. Further inquiries can be directed to the corresponding author.

## Abbreviations

The following abbreviations are used in this manuscript:

PVC	Premature Ventricular Contraction
